# Complementary and alternative medicine (CAM) as part of primary health care in Germany–comparison of patients consulting general practitioners and CAM practitioners: a cross-sectional study

**DOI:** 10.1186/s12906-016-1402-8

**Published:** 2016-10-24

**Authors:** Katja Krug, Katharina I. Kraus, Kathrin Herrmann, Stefanie Joos

**Affiliations:** 1 Department of General Practice and Health Services Research, University Hospital Heidelberg, Marsilius-Arkaden, Turm West, Im Neuenheimer Feld 130.3, 69120 Heidelberg, Germany; 2 Institute of General Practice and Interprofessional Care, University Hospital Tübingen, Österbergstr. 9, 72074 Tübingen, Germany

**Keywords:** Primary health care, Complementary medicine, General practice, Reasons for encounter, Patient characteristics

## Abstract

**Background:**

In Germany, complementary and alternative medicine (CAM) in primary health care is offered by general practitioners (GPs) and by natural health practitioners, so called ‘Heilpraktiker’ (HPs). Considering the steadily growing number of unregulated HPs, the aim of the study was to assess characteristics of patients consulting HPs in comparison to patients consulting GPs.

**Methods:**

In a cross-sectional study, patients of randomly selected GPs and HPs were asked to complete a questionnaire about their health care status, health care behavior, and symptoms rated on the Measure Yourself Medical Outcome Profile (MYMOP-D). Patient groups were compared based on health care provider (HP, GP with high use of CAM (CAM-GP), and GP with no/little use of CAM (nCAM-GP)) using Kruskal-Wallis tests and analyses of variance (ANOVA).

**Results:**

Altogether, 567 patients (91 of 11 HPs, 223 of 15 CAM-GPs, 253 of 19 nCAM-GPs) filled in the questionnaire. Patients of HPs had a higher education level and were more often female. The most common reason for encounter among all three groups were musculoskeletal problems (30.2–31.1 %). Patients seeing HPs reported more psychological (4.4 % vs. 17.8 %), but less respiratory problems (19.9 % vs. 7.8 %), and longer symptom duration (>5 years: 21.1 % vs. 40.7 %), than patients of nCAM-GPs.

**Conclusions:**

The high percentage of patients with psychological illness and chronic health problems consulting HPs demonstrates the urgent need for action with regard to CAM therapy in primary care and regulation of natural health practitioners. Appropriate measures with regard to quality and patient safety should be taken given the growing numbers of HPs and the absence of a regulatory body.

**Electronic supplementary material:**

The online version of this article (doi:10.1186/s12906-016-1402-8) contains supplementary material, which is available to authorized users.

## Background

Complementary and Alternative Medicine (CAM) has become increasingly accepted in Western countries in recent decades [1], nevertheless CAM therapies are not without potential risk. For example, interactions between standard medications and CAM interventions have been shown [2]. However, the fact that patients do not necessarily inform health care professionals of their CAM use poses a concern for patient safety [3]. With a better understanding of the characteristics of patients that chose CAM, quality care outcomes can be ensured. In the German health service context, an important further issue related to CAM with potential impact on patient safety is that complementary and alternative therapies can be offered by unregulated natural health practitioners as well as by regulated health professionals (primarily medical doctors). It is important to gain information about patient characteristics, consultation patterns and patient outcomes. Answers to such questions as: Which patients are consulting CAM practitioners, with which health symptoms? Our study examined reason for encounter in the primary care context, with a focus on reviewing characteristics of patients consulting unregulated natural health practitioners in comparison to those consulting general practitioners (GPs) offering CAM. This is relevant in the first instance for patient safety but also with regard to further development of evidence-based primary health care services for patients.

In Germany, there is a state recognized natural health practitioner role called the *Heilpraktiker* (HP). The legal practice of HPs was established in the late 1930s [4] but this group are not regulated health professionals in the traditional sense. To “qualify”, HPs have to pass an exam on basic medical knowledge and skills at a local public health office to obtain initial state recognition and in order to “exclude danger to the health of the nation” [4]. No formal medical training is required and there is no compulsory curriculum for schooling, advanced training or achieving practical skills. HPs have free scope to perform a range of clinical interventions on their patients, with the exception of gynecology, dentistry, prescription of medication and treating infectious diseases. They provide a great variety of CAM therapies, both invasive and non-invasive. Only those HPs performing invasive procedures, i.e. injections or acupuncture, are under intermittent surveillance by the local public health offices under the Infection Protection Act (2000). To date, there have been only few scientific studies investigating health services provided by HPs or experiences of patients with HPs in Germany [5, 6].

In Germany, many medical doctors also provide CAM [6], especially GPs [7]. Through successful completion of further education, they can qualify in CAM disciplines, e.g. naturopathy, chiropractic, homeopathy, acupuncture, which are accredited by the German Medical Council. According to the Federal Health Monitoring, in 2015, among 485.00 doctors [8], more than 67.000 had CAM qualifications [9]. However, many GPs are additionally providing CAM in their daily practice without having a formally recognised CAM qualification. A survey in 2009 revealed, about 60 % of all GPs in Germany provide CAM [10]. It is known that patients deliberately choose to use CAM [3], e.g. to avoid side effects from pharmaceutical drugs. Therefore, natural health practitioners and medical doctors offering CAM might actually complement orthodox medical approaches.

At present, in Germany very few CAM interventions are reimbursed under statutory health insurance (SHI), and when, then only when patients consult a physician holding the corresponding CAM qualification. Consultations by HPs are not covered by SHI and have to be paid out-of-pocket by patients. However, most private health insurance companies will reimburse payments made for services provided by HPs.

Despite comparatively high numbers of HPs in Germany (40.000 in 2014 [11], compared with 58.000 GPs in 2014 [8]), there is nearly no research in this field and consequently, we know little about consultation patterns, patient characteristics and patient outcomes when consulting HPs. A few studies have elaborated on patient characteristics for the motivation of CAM use, thus identifying psychosocial factors as age, educational level and health behaviour as influencing CAM use [12]. International studies on the role of CAM in primary healthcare, especially in terms of natural health practitioners, are inconsistent [13, 14]. This suggests substantial influences on acceptance are due to culture and tradition as well as to differences in national health systems. Therefore, the aim of our study was to assess patient characteristics including health behaviour and consultation patterns of patients consulting HPs in comparison to patients consulting GPs as a means of producing evidence in this relevant area of primary health care.

## Methods

We conducted a cross-sectional study by means of paper-based questionnaires.

### Questionnaire

The questionnaire comprised validated instruments and single item questions for sociodemographic data and comorbid conditions (Additional file 1). As indicators for health behavior, smoking status, physical activity and self-evaluation of attention to own health were assessed. The questionnaire also included a question on consulting (other) HPs and/or (other) physicians, of whom it was not assessed if they offered CAM, for the same health problem.

The reasons for consultations were assessed by the Measure Yourself Medical Outcome Profile (MYMOP) [15, 16]. Within the MYMOP, patients define 1 or 2 main symptoms as free text. In further questions, they have to rank the severity of these self-defined symptoms on a 7-point Likert-scale with 0 as best and 6 as worst and classify the duration of their symptoms. Furthermore, there are questions concerning their general wellbeing, restriction of an activity of individual choice due to the symptoms, and the treatment they already received or their expectation towards future treatment. A MYMOP Profile Score can be calculated as the mean of the ratings for severity of main symptoms, general wellbeing and restriction of activity. The German version of the MYMOP (MYMOP-D) has been validated within this study [17].

GPs and HPs received a questionnaire consisting of sociodemographic questions regarding age, practice location, duration of practice and use of CAM in their practices.

### Sample and recruitment

We intended to recruit 600 patients in 30–40 practices of GPs (to be divided in high- and low-/no users of CAM) and 10–20 practices of HPs (i.e. approximately 5–15 patients per practice) in the south of Germany. We invited 153 HPs and 931 GPs by mail to participate based on random selection from the telephone book (HPs) and from a publicly accessible list of the German Medical Association in Baden-Württemberg (www.kvbawue.de). We explicitly aimed to include GPs with and without additional CAM qualifications. All adult patients who were scheduled to consult a GP/HP during pre-defined consultation hours were verbally invited to take part in the study (convenience sample). Patients with cognitive limitations (e.g. dementia), language problems and those consulting GPs only for organizational reasons (e.g. repeat of a prescription) or check-ups were excluded from the study. All interested providers and patients received oral and written information about the study. No information was gathered before receiving written consent.

### Data collection

In the participating GPs’ practices, data collection took place during normal consultation hours. Two doctoral students (KIK, KH) invited all patients within this time period in the waiting room to take part in the study. Interested patients received the questionnaire and, if necessary, they were supported in completing the questionnaire. Completed questionnaires were collected immediately by KIK and KH. When the required minimum number of five patients per practice was not reached on 1 day, one further appointment in the practice was made.

In the HPs’ practices, this procedure was not feasible because many HPs had only a few consultations per day. Consequently, HPs were briefed personally on the patient questionnaires and asked to hand them out consecutively to all patients within up to 6 months.

### Data analysis

Sociodemographic data and questionnaire scores are reported as frequencies and means with standard deviation. For the analyses of consultation patterns, the patients’ first main symptoms assessed by the MYMOP-D were post hoc classified according to the ICPC2 (International Classification of Primary Care) [18].

GPs were divided according to their self-reported CAM use: nCAM-GPs (no CAM use/missing (*n* = 7) or <30 % CAM use (*n* = 12)) and CAM-GPs (≥30 % CAM use; *n* = 15).

Chi-square tests were used to analyze differences in frequencies of ICPC2 classes, sociodemographics, smoking status, and consultation of (other) physicians and/or HPs between patients consulting HPs, CAM-GPs and nCAM-GPs. Kruskal-Wallis tests were used to analyze differences in education, duration of complaints and physical activity. Oneway ANOVA was used to analyze differences in sum scores of the MYMOP-D, attention to health, severity of complaints and number of chronic diseases. All valid data per test were included in the individual tests to reach the maximum sample size per test. Statistical significance was set at *p* < .05. For all statistical analyses IBM SPSS 20 (IBM Corp.) was used.

## Results

Thirty-four GPs (19 female, 14 male; age: M = 51.2, SD = 8.2; professional experience in years: M = 21.7, SD = 7.9; *n* = 1 data missing) and 11 HPs (9 female, 1 male; age: M = 49.7, SD = 6.4; professional experience in years: M = 14.6, SD = 7.9; *n* = 1 data missing) took part in the study. “Heilpraktiker” was the primary occupation of 9 HPs (4 full-time, 5 part-time). GPs and HPs offered a wide range of CAM therapies, although GPs more often offered evidence-based methods (i.e. acupuncture, herbalism) (Table 1). GPs additionally had a variety of accredited qualifications, i.e. in acupuncture (*n* = 7), naturopathy (*n* = 14), emergency medicine (*n* = 7), pain therapy (*n* = 3), sports medicine (*n* = 3), manual therapy (*n* = 2), and homeopathy (*n* = 4).

**Table 1:** CAM methods offered by “Heilpraktiker” (HPs) and general practitioners (GPs)

	HPs (*n*=10)	GPs (*n*=33)
Acupuncture	6	13
Anthroposophic medicine	1	2
Autohemotherapy		5
Biological medicine	2	2
Cupping therapy	1	1
Herbalism	3	10
Homeopathy	9	13
Hypnotherapy	1	1
Massage	2	
Naturopathy		4
Neural therapy		5
Orthomolecular medicine		2
Osteopathy	2	
Traditional Chinese medicine	1	1
others	8 (i.e. chromotherapy, craniosacral therapy, magnet therapy, manual therapy, reflexology, thought field therapy)	8 (i.e. applied kinesiology, athletic taping, bioresonance, hyperthermia, leeches)

The mean patient number recruited per practice was 14 (min 3; max 18) for the GP practices and 8 (min 1; max 15) for the HP practices. Altogether, 567 patients were included: 91 HP patients and 476 GP patients, thereof 223 (46.8 %) were consulting a CAM-GP and 253 (53.2 %) a nCAM-GP.

### Characteristics of patients

#### Sociodemographics

While age was comparable between patients of the three provider groups (HPs, CAM-GPs and nCAM-GPs), significant differences (*p*< .05) regarding gender, education, marital status, country of birth and health insurance status could be seen (Table 2).

**Table 2:** Patient characteristics

	GP patients			HP patients (*n*=91)	*p* ^b^
	Total (*n*=476)	nCAM-GP (*n*=253)	CAM-GP (*n*=223)		
Age mean (years)	50.1 (SD 18.4)	51.2 (SD 19.3)	48.9 (SD 17.2)	49.7 (SD 15.0)	.36
Sex ratio (*n*, %) F:M	293:183 (61.6:38.4)	140:113 (55.3:44.7)	153:70 (68.6:31.4)	75:16 (82.4:17.6)	<.01
Highest level of education (*n*; %^a^)					
Up to secondary modern school or other	148 (31.4)	87 (34.8)	61 (27.5)	15 (16.5)	.02
Junior high school	125 (26.5)	66 (26.4)	59 (26.6)	32 (35.2)	
University-entrance diploma	199 (42.2)	97 (38.8)	102 (45.9)	44 (48.4)	
Marital status (*n*; %^a^)					
Unmarried	131 (27.7)	63 (25.1)	68 (30.6)	13 (14.3)	.01
Married/in a partnership	241 (51.0)	127 (50.6)	114 (51.4)	62 (68.1)	
Divorced /separated	51 (10.8)	28 (11.2)	23 (10.4)	10 (11.0)	
Widowed	50 (10.6)	33 (13.1)	17 (7.7)	6 (6.6%)	
Country of birth (*n*; %^a^)					
Germany	391 (82.7)	215 (85.7)	176 (79.3)	84 (92.3)	.01
Other country	82 (17.3)	36 (14.3)	46 (20.7)	7 (7.7)	
Language primarily spoken at home (*n*; %^a^)					
German	432 889.4)	225 (89.6)	198 (89.2)	88 (96.7)	.26
Other language	25 (5.3)	12 (4.8)	13 (5.9)	2 (2.2)	
No specification possible	25 (5.3)	14 (5.6)	11 (5.0)	1 (1.1)	
Statutory health insurance (*n*; %^a^)	426 (89.7)	286 (90.5)	198 (88.8)	66 (72.5)	<.01

^a^numbers do not add up to *n*=567 due to missing values, ^b^significance of difference between the three groups

#### Comorbidities and health behavior

Patients suffered from up to 7 (on average 1.6) comorbid conditions. No significant difference was found between patient groups. Highly significant differences were observed in smoking status, physical activity and self-evaluation of attention to own health pointing towards a more favorable profile in HP and CAM-GP patients in comparison to nCAM-GPs (Table 3). HP patients more often consulted other physicians, including GPs, and additional HPs compared to GP patients (Table 2).

**Table 3:** Health behavior of patients

	GP patients			HP patients	*p* ^c^
	Total	nCAM-GP	CAM-GP		
“Do you smoke?” (*n*; %^a^)					
No	298 (62.7)	155 (61.5)	143 (64.1)	66 (72.5)	<.01
Former smoker	33 (6.9)	17 (6.7)	16 (7.2)	12 (13.2)	
Occasionally	60 (12.6)	27 (10.7)	33 (14.8)	7 (7.7)	
Yes	84 (17.7)	53 (21.0)	31 (13.9)	6 (6.6)	
“How often are you physically active?” (*n*; %^a^)					
Never	120 (25.6)	74 (29.5)	46 (21.2)	9 (9.9)	<.01
Once a week or less	163 (34.8)	90 (35.9)	73 (33.6)	37 (40.7)	
Few times a week	172 (36.8)	81 (32.3)	91 (41.9)	41 (45.1)	
Daily	13 (2.8)	6 (2.4)	7 (3.2)	4 (4.4)	
“How much attention do you pay to your health?” M (SD)^b^					
	4.3 (1.4)	4.1 (1.4)	4.6 (1.3)	4.7 (1.1)	<.01
Consultation of (other) physicians (*n*; %^a^)					
Yes	217 (45.7)	101 (39.9)	116 (52.3)	76 (83.5)	<.01
No	258 (54.3)	152 (60.1)	106 (47.7)	15 (16.5)	
Consultation of (other) GPs (*n*; %*)					
Yes	22 (4.6)	10 (4.0)	12 (5.4)	19 (20.9)	<.01
No	453 (95.4)	243 (96.0)	210 (94.6)	72 (79.1)	
Consultation of (other) HPs (*n*; %*)					
Yes	36 (7.6)	13 (5.1)	23 (10.4)	26 (28.6)	<.01
No	439 (92.4)	240 (94.9)	199 (89.6)	65 (71.4)	

^a^numbers do not add up to *n*=567 due to missing values; ^b^7-point-Likert-scale (0 = “not at all” … 6 = “very much”), ^c^significance of difference between the three groups

### Main symptoms

**Fig. 1:**
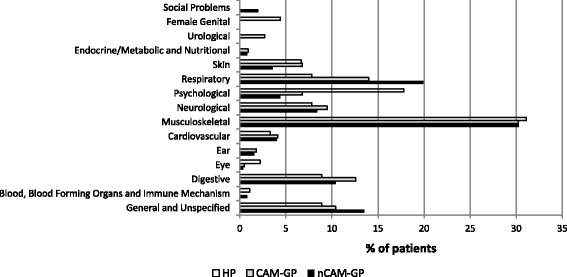
First main symptoms given in the MYMOP, classified in categories of the International Classification for Primary Care (ICPC-2) (nCAM-GP: GP with no/little use of CAM, CAM-GP: GP with at least 30 % use of CAM, HP: non-medical CAM practitioner)

For the majority of patients, the symptom given first referred to the musculoskeletal system (nCAM-GP: 30.3 %, CAM-GP 30.2 %; HP: 31.1 %) (Fig. 1). However, HP patients reported more psychological problems (nCAM-GP: 4.4 %, CAM-GP: 6.8 %; HP: 17.8 %) and less respiratory problems (nCAM-GP: 19.9 %, CAM-GP 14.0 %; HP 7.8 %).

#### Duration and severity of symptoms

**Fig. 2:**
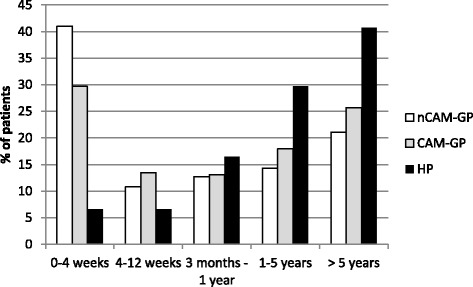
Duration of symptoms (nCAM-GP: GP with no/little use of CAM, CAM-GP: GP with at least 30% use of CAM, HP: non-medical CAM practitioner)

More than 40 % of patients consulting HPs experienced their main symptoms for >5 years compared to 21.1 % of the nCAM-GPs’ patients and 25.7 % of the CAM-GPs’ patients (*p* < .01) (Fig. 2).

**Fig. 3:**
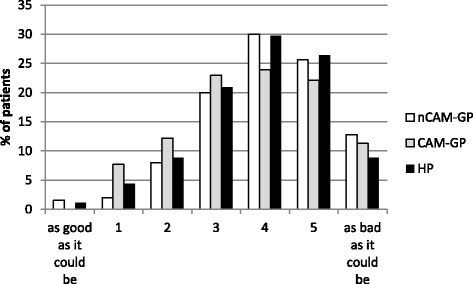
Severity of symptoms (nCAM-GP: GP with no/little use of CAM, CAM-GP: GP with at least 30% use of CAM, HP: non-medical CAM practitioner)

In contrast, symptom severity showed only few differences comparing ratings among the three patient groups (nCAM-GP M = 4.0, SD = 1.3; CAM-GP M = 3.7, SD = 1.4; HP M = 3.9, SD = 1.4, *p* = .08) (Fig. 3).

## Discussion

To our knowledge, this was the first study assessing patient characteristics including health behavior and consultation patterns of patients consulting HPs in comparison to patients consulting GPs in Germany. We found similarities but also a series of relevant differences between patients cared for by the two GP groups (with low and high use of CAM) and patients of HPs. Significantly more women and patients with psychological problems consult HPs compared to GPs. Furthermore, patients seeing HPs seemed to have more favorable health behavior. Among HP patients, we found a considerably higher proportion of patients with long term complaints. Altogether, our results indicate that characteristics and consultation patterns of HP patients are different from those of GP patients, and GPs providing CAM seem to have an “in-between status”.

Our results are in alignment with studies from the UK, which have shown that a large number of patients consulting CAM practitioners have musculoskeletal problems [13, 19]. Our results clearly show that this was a main symptom among HP patients as a typical reason for encounter within primary health care. The only differences found across the three practitioner groups were for psychological problems and respiratory diseases. A simple explanation for the latter might be that HPs are not allowed to provide medical certificates for sick leave for patients. One of the most frequent reasons for acute consultations in German GP practices and for medical certificates for sick leave are respiratory complaints as symptoms of cold [20].

HPs were considerably more frequently consulted for long-term problems compared to GPs, a circumstance that has already been shown by Paterson two decades ago [13]. The higher percentage of HP patients with long-term diseases might be due to dissatisfaction with GP care in the course of the disease. Or maybe HPs are seen as a “last resort” when patients fail to recognize improvements with the orthodox approach offered by physicians. Still, the self-reported severity of the symptoms did not differ between patients cared for by HPs or GPs. Additionally, HP patients more often consulted other physicians and HPs and might be especially frequent users of health care services.

The high percentage of psychological symptoms among the HP patient group was surprising. Reasons may be that patients consulting a HP hope for more time to talk about their problems at “eye-level” or fear of getting a stigmatizing diagnosis when they go to a physician. In one review, we found a high usage of CAM among patients with depressive disorders was described [21].

In Germany, patients with all kinds of symptoms may consult a general practitioner. In our study, GPs offering CAM therapies and HPs did not differ with respect to the broad spectrum of therapies offered, although GPs more often provided evidence-based methods like acupuncture and herbalism, which are covered by statutory health insurance. To elaborate on reasons why patients go to one provider or another who offers the same intervention, studies with a qualitative approach are needed.

Previous studies have been inconsistent with regard to gender: while the higher probability that women use CAM is well known and described in several studies [22–24], in the study of Paterson [13] gender and age did not differ significantly between patients consulting CAM practitioners and physicians. In our study, the gender distribution on the patient side is mirrored in the professional side: more female health practitioners also offer CAM (either as HPs or CAM-GPs).

Contrary to the findings of a large German study of acupuncture users among internal medicine patients [25], our patients using CAM did not differ in age from non-CAM users, whereas the percentage of current smokers was also highest in patients of nCAM-GPs; the HP patients in our study were less often currently smoking and more physically active. The observed positive relationship between CAM use and health behavior was also shown in a study with childhood cancer survivors [26]. The relationship between higher education and higher CAM use was also described in an international review [23] and in a population-based German study [27], although the relationship was weaker in the patients of our study.

### Limitations

Although our study has gathered exploratory information about consulting patterns and wishes of patients, the different methods of data collection in GP and HP practices might have influenced the results. However, there were only a few questions asking for a subjective judgment of the patients, most questions were related to quantifiable aspects.

Additionally, the participation rates of GPs, HPs, and their patients were generally lower than expected. Due to logistic reasons, we were not able to recruit more participants. Nevertheless, this was the first study assessing patients in the setting of the HP practice.

## Conclusions

In conclusion, the high percentage of patients with psychological and chronic problems consulting HPs with the hope to find help in CAM should provoke decision-makers and health care policy makers to think about the appropriateness and effectiveness of regulation of natural health practitioners in the current German health care system. Our results demonstrate the urgent need for action considering the steadily growing numbers of HPs (nearly comparable to that of GPs) despite of a lacking compulsory curriculum and medical training.

## Additional file

Additional file 1: Patient questionnaire used in the study. (PDF 146 kb)

## Abbreviations

CAM: Complementary and alternative medicine
GP: General practitioner
HP: *Heilpraktiker*
ICPC: International classification of primary care
MYMOP: Measure yourself medical outcome profile
SHI: Statutory health insurance
