# Supplementary material for: Complementary and alternative medicine (CAM) as part of primary health care in Germany–comparison of patients consulting general practitioners and CAM practitioners: a cross-sectional study
Source: BMC Complement Altern Med. 2016 Oct 24;16:409. doi: 10.1186/s12906-016-1402-8 (PMC5078911; doi:10.1186/s12906-016-1402-8)
Supplement: Additional file 1: — Patient questionnaire used in the study. (PDF 146 kb) [file 12906_2016_1402_MOESM1_ESM.pdf]

UniversitätsKlinikum Heidelberg

KompMed Primär-Studie

Patientenfragebogen A

Version 16.2.2012

Bitte beachten Sie beim Ausfüllen folgende **Hinweise**:

- *Verwenden Sie nur Kugelschreiber.*
- *Machen Sie Freitextangaben bitte in Druckbuchstaben.*
- *Kreuzen Sie die Kästchen deutlich an:* X
- *Bei versehentlicher Falschauswahl füllen Sie bitte das entsprechende Kästchen ganz aus* ■ *und kreuzen Ihre Auswahl erneut an:* X

A. Fragen zu Ihrem Gesundheitszustand

Name: ____ Telnr: ______ /

Geburtsdatum: __ __ . __ __ . __ __ __ __ ______________

Straße: ____ Hausnr.: ______

Postleitzahl: __ __ __ __ __

Ort: ____

Datum der heutigen Befragung: __ __ . __ __ . __ __ __ __

Behandelnder Arzt: ____

Bitte machen Sie jeweils ein Kreuz (X) auf der für Sie zutreffenden Zahl:

1.) Wählen Sie bitte **ein oder zwei Beschwerden** (körperlich oder geistig), die Sie am meisten stören. Schreiben Sie diese in das dafür vorgesehene Feld. Überlegen Sie sich nun sorgfältig, wie ausgeprägt Sie jede Beschwerde in der vergangenen Woche empfunden haben und machen Sie ein Kreuz auf der dafür zutreffenden Zahl.

Beschwerde 1: ______________________________

So gut, wie es nur sein könnte — So schlecht, wie es nur sein könnte

☐ 0 ☐ 1 ☐ 2 ☐ 3 ☐ 4 ☐ 5 ☐ 6

Beschwerde 2: ______________________________

So gut, wie es nur sein könnte — So schlecht, wie es nur sein könnte

☐ 0 ☐ 1 ☐ 2 ☐ 3 ☐ 4 ☐ 5 ☐ 6

2.) Wählen Sie jetzt **eine Aktivität** (körperlich, sozial oder geistig), die Ihnen wichtig ist, und deren Ausübung durch Ihre Beschwerde(n) erschwert bzw. gänzlich eingeschränkt wird. Kreuzen Sie bitte an, wie ausgeprägt dies in der vergangenen Woche war.

Aktivität: ______________________________

Gar nicht eingeschränkt — Gänzlich eingeschränkt

☐ 0 ☐ 1 ☐ 2 ☐ 3 ☐ 4 ☐ 5 ☐ 6

Kontakt: PD Dr. med. Stefanie Joos, Universitätsklinikum Heidelberg, Abteilung Allgemeinmedizin und Versorgungsforschung
Voßstr. 2, 69115 Heidelberg, Telefon: 06221/56-6263, Fax: 06221/56-1972

3.) Allgemeines Wohlergehen:

Wie würden Sie Ihr allgemeines Befinden in der vergangenen Woche bewerten?

So gut, wie es nur sein könnte						So schlecht, wie es nur sein könnte
☐	☐	☐	☐	☐	☐	☐
0	1	2	3	4	5	6

4.) Wie lange leiden Sie bereits **unter Beschwerde 1**, egal ob Sie durchgehend oder gelegentlich daran gelitten haben? Bitte kreuzen Sie an.

☐ 0-4 Wochen ☐ 4-12 Wochen ☐ 3 Monate – 1 Jahr ☐ 1 – 5 Jahre ☐ über 5 Jahre

---

5.) Nehmen Sie **Medikamente** wegen dieser Beschwerden?

☐ Ja ☐ Nein

falls JA:

5a) Bitte schreiben Sie die Namen der Medikamente auf und wie oft Sie diese pro Tag einnehmen:

____

____

5b) Ist es wichtig für Sie, diese Medikamente zu reduzieren?

☐ nicht wichtig ☐ ein bisschen wichtig ☐ sehr wichtig ☐ keine Angabe

falls NEIN:

5c) Wie wichtig ist es für Sie, auch in Zukunft keine Medikamente gegen diese Beschwerde(n) zu nehmen?

☐ nicht wichtig ☐ ein bisschen wichtig ☐ sehr wichtig ☐ keine Angabe

---

6.) Sind Sie wegen dieser Beschwerde(n) schon bei einem anderen Arzt / Therapeuten gewesen?

☐ nein

☐ ja Wenn ja, bei einem ☐ Facharzt für ___________________ *(bitte eintragen)*

.

☐ Heilpraktiker

☐ sonstige: _____________________ *(bitte eintragen)*

7.) Haben Sie eine chronische Krankheit?

☐ **Nein,** ich leide unter keiner chronischen Erkrankung.

Ja, *(bitte die entsprechende Erkrankung ankreuzen; Mehrfachantworten möglich)*

☐ chronische Rückenschmerzen
☐ Gelenkerkrankung (Arthrose, Rheuma)
☐ Allergie / chronische Hauterkrankung
☐ Koronare Herzerkrankung / Herzschwäche
☐ Bluthochdruck (Hypertonie)
☐ Diabetes mellitus (Zuckerkrankheit)
☐ Asthma oder Chronische Bronchitis
☐ chronische Magen-Darm-Krankheit
☐ Depression / Angsterkrankung
☐ Krebserkrankung

Wenn ja, welche Krebserkrankung: |_|_|_|_|_|_|_|_|_|_|_|_|_|_|_|_|

☐ sonstige chronische Erkrankungen

Wenn ja, welche |_|_|_|_|_|_|_|_|_|_|_|_|_|_|_|_|_|_|_|_|

8.) Wieviele Medikamente nehmen Sie insgesamt ein (incl. eventueller pflanzlicher, homöopathischer Präparate)?

☐ keine ☐ 1-2 ☐ 3-4 ☐ 5-6 ☐ >7

9.) Bitte geben Sie an, welche Aussagen Ihren heutigen Gesundheitszustand am besten beschreiben, indem Sie ein Kreuz in ein Kästchen jeder Gruppe machen.

a.) Beweglichkeit/Mobilität

Ich habe keine Probleme herumzugehen ☐
Ich habe einige Probleme herumzugehen ☐
Ich bin ans Bett gebunden ☐

b.) Für sich selbst sorgen

Ich habe keine Probleme, für mich selbst zu sorgen ☐
Ich habe einige Probleme, mich selbst zu waschen oder mich anzuziehen ☐
Ich bin nicht in der Lage, mich selbst zu waschen oder anzuziehen ☐

c.) Alltägliche Tätigkeiten *(z.B. Arbeit, Hausarbeit, Familien- oder Freizeitaktivitäten)*

Ich habe keine Probleme, meinen alltäglichen Tätigkeiten nachzugehen ☐
Ich habe einige Probleme, meinen alltäglichen Tätigkeiten nachzugehen ☐
Ich bin nicht in der Lage, meinen alltäglichen Tätigkeiten nachzugehen ☐

Kontakt: PD Dr. med. Stefanie Joos, Universitätsklinikum Heidelberg, Abteilung Allgemeinmedizin und Versorgungsforschung
Voßstr. 2, 69115 Heidelberg, Telefon: 06221/56-6263, Fax: 06221/56-1972

d.) Schmerzen/Körperliche Beschwerden

Ich habe keine Schmerzen oder Beschwerden ☐

Ich habe mäßige Schmerzen oder Beschwerden ☐

Ich habe extreme Schmerzen oder Beschwerden ☐

e.) Angst/Niedergeschlagenheit

Ich bin nicht ängstlich oder deprimiert ☐

Ich bin mäßig ängstlich oder deprimiert ☐

Ich bin extrem ängstlich oder deprimiert ☐

10.) Bitte schätzen Sie Ihren Gesundheitszustand ein.

Um Sie bei der Einschätzung, wie gut oder wie schlecht Ihr Gesundheitszustand ist, zu unterstützen, haben wir eine Skala gezeichnet, ähnlich einem Thermometer. Der best denkbare Gesundheitszustand ist mit einer "100" gekennzeichnet, der schlechteste mit "0".

Wir möchten Sie nun bitten, auf dieser Skala zu kennzeichnen, wie gut oder schlecht Ihrer Ansicht nach Ihr persönlicher Gesundheitszustand heute ist.

Bitte markieren Sie dazu das Thermometer an der für Sie passenden Stelle mit einem Kreuz.

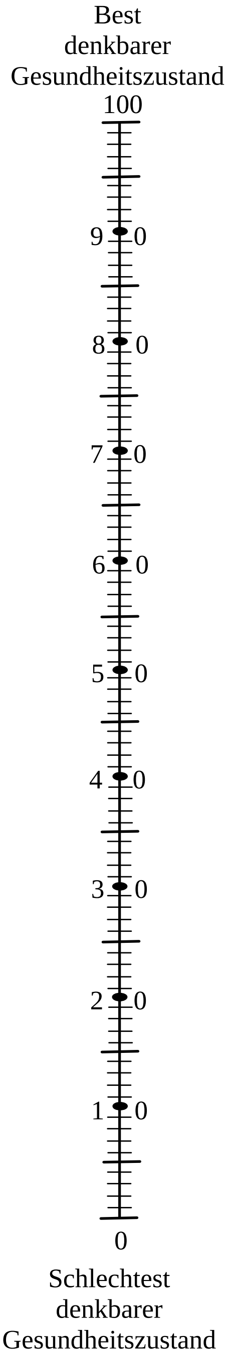

Kontakt: PD Dr. med. Stefanie Joos, Universitätsklinikum Heidelberg, Abteilung Allgemeinmedizin und Versorgungsforschung
Voßstr. 2, 69115 Heidelberg, Telefon: 06221/56-6263, Fax: 06221/56-1972

B. Fragen zu Ihrem Arzt und zur Konsultation

Die folgenden Fragen beziehen sich alle auf den Arzt, bei dem Sie heute waren.

11.) Wie lange kennen Sie Ihren Arzt bereits?

☐ Es ist meine erste Konsultation
☐ weniger als 1 Jahr ☐ seit 1-5 Jahren
☐ seit 5-10 Jahren ☐ länger als 10 Jahre

12.) Wie oft waren Sie (abgesehen vom heutigen Besuch) innerhalb der letzten 12 Monate bei Ihrem Arzt?

☐ nie ☐ weniger als 5-mal ☐ 5 bis 10-mal ☐ mehr als 10-mal

13.) Ich kann mit meinem Arzt offen sprechen.

stimme überhaupt nicht zu						stimme voll zu
☐	☐	☐	☐	☐	☐	☐

14.) Ich habe volles Vertrauen in meinen Arzt.

stimme überhaupt nicht zu						stimme voll zu
☐	☐	☐	☐	☐	☐	☐

15.) Wie wohl fühlen Sie sich in der Praxis insgesamt (Atmosphäre, Praxisteam, Ausstattung u.a.)**?**

überhaupt nicht wohl						sehr wohl
☐	☐	☐	☐	☐	☐	☐

16.) Würden Sie Ihren Arzt Ihrer Familie / Ihren Freunden empfehlen?

überhaupt nicht empfehlen						in jedem Fall empfehlen
☐	☐	☐	☐	☐	☐	☐

17.) Wie gestresst schätzen Sie Ihren Arzt <u>heute</u> ein?

Bitte kreisen Sie am Thermometer die Zahl ein (0-10), die das am Besten beschreibt.

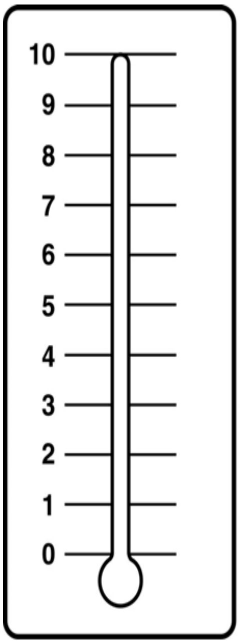

sehr gestresst

nicht gestresst

Kontakt:
PD Dr. med. Stefanie Joos, Universitätsklinikum Heidelberg, Abteilung Allgemeinmedizin und Versorgungsforschung
Voßstr. 2, 69115 Heidelberg, Telefon: 06221/56-6263, Fax: 06221/56-1972

Bitte bewerten Sie die folgenden Aussagen in Bezug auf Ihre heutige Konsultation:

	trifft überhaupt nicht zu				trifft voll zu	keine Angabe
**18.) Hat sich der Arzt so verhalten, dass Sie sich in seiner Nähe wohlfühlen konnten?** (Er war freundlich, warmherzig und respektvoll, aber nicht kühl oder kurz angebunden.)	☐	☐	☐	☐	☐	☐
**19.) Hat der Arzt Sie Ihre eigene (Krankheits-) Geschichte erzählen lassen?** (Er gab Ihnen Zeit, Ihre Krankheit ausführlich zu beschreiben. Er hat Sie dabei nicht unterbrochen oder abgelenkt.)	☐	☐	☐	☐	☐	☐
**20.) Hat der Arzt Ihnen wirklich zugehört?** (Er hat dem, was Sie gesagt haben, seine volle Aufmerksamkeit geschenkt und dabei nicht auf seine Unterlagen oder auf den Computer geschaut.)	☐	☐	☐	☐	☐	☐
**21.) Hat sich der Arzt für Sie als Mensch und für Ihr Umfeld interessiert?** (Er kannte oder fragte nach wichtigen Einzelheiten Ihres Lebens oder Ihrer persönlichen Situation und hat Sie nicht wie eine „Nummer“ behandelt.)	☐	☐	☐	☐	☐	☐
**22.) Hat der Arzt Ihre Sorgen wirklich verstanden?** (Er konnte Ihnen das Gefühl vermitteln, dass er Ihre Sorgen genau verstanden hat. Er hat dabei nichts übersehen und ist über nichts hinweggegangen.)	☐	☐	☐	☐	☐	☐
**23.) War der Arzt fürsorglich und hat er Mitgefühl gezeigt?** (Er hat sich aufrichtig um Sie gekümmert und sich Ihnen gegenüber menschlich gezeigt. Dabei war er nicht gleichgültig oder distanziert.)	☐	☐	☐	☐	☐	☐
**24.) Hat der Arzt Ihnen Mut gemacht?** (Er hatte eine zuversichtliche Einstellung. Er war ehrlich, aber gegenüber Ihren Problemen nicht negativ eingestellt.)	☐	☐	☐	☐	☐	☐
**25.) Hat der Arzt Ihnen alles verständlich erklärt?** (Er hat Ihre Fragen vollständig beantwortet und alles eindeutig erklärt. Er gab Ihnen ausreichende Informationen und hat Sie nicht im Unklaren gelassen.)	☐	☐	☐	☐	☐	☐
**26.) Hat der Arzt Ihnen geholfen, einen Weg zu finden mit Ihrer Krankheit umzugehen?** (Er hat gemeinsam mit Ihnen erkundet, wie Sie selbst Ihren Gesundheitszustand verbessern können. Dabei hat er Sie ermutigt, anstatt Sie zu belehren.)	☐	☐	☐	☐	☐	☐
**27.) Hat der Arzt mit Ihnen zusammen einen Behandlungsplan erstellt?** (Er hat mit Ihnen die Behandlungsmöglichkeiten diskutiert und Sie in Entscheidungen – soweit Sie dies gewünscht haben - einbezogen. Dabei hat er Ihre Sichtweise nicht ignoriert.)	☐	☐	☐	☐	☐	☐

Kontakt:
PD Dr. med. Stefanie Joos, Universitätsklinikum Heidelberg, Abteilung Allgemeinmedizin und Versorgungsforschung
Voßstr. 2, 69115 Heidelberg, Telefon: 06221/56-6263, Fax: 06221/56-1972

28.) Wie zufrieden sind Sie insgesamt mit der heutigen Konsultation?

überhaupt nicht zufrieden						sehr zufrieden
☐	☐	☐	☐	☐	☐	☐

29.) Wie lange hat Ihre heutige Konsultation gedauert? *(Bitte schätzen Sie.)*

- ☐ weniger als 5 Minuten
- ☐ 5 bis 10 Minuten
- ☐ 10 bis 15 Minuten
- ☐ 15 bis 20 Minuten
- ☐ 20 bis 40 Minuten
- ☐ 40 bis 60 Minuten
- ☐ länger als 60 Minuten

30.) Die Länge der Konsultation war ....

- ☐ ... genau richtig
- ☐ ... zu kurz
- ☐ ... zu lang

31.) Fühlen Sie sich durch Ihren heutigen Besuch beim Arzt in der Lage …

Bitte kreuzen Sie in jeder Zeile nur eine Antwortmöglichkeit an!

	viel besser	etwas besser	unver-ändert	etwas schlechter	viel schlechter	keine Angabe
**a.)** … mit Ihrem Leben umzugehen?	☐	☐	☐	☐	☐	☐
**b.)** … Ihre Krankheit zu verstehen?	☐	☐	☐	☐	☐	☐
**c.)** … mit Ihrer Krankheit umzugehen?	☐	☐	☐	☐	☐	☐
**d.)** … selbst etwas für Ihre Gesundheit/ gegen Ihre Krankheit tun zu können?	☐	☐	☐	☐	☐	☐

	viel mehr	etwas mehr	unver-ändert	etwas weniger	viel weniger	keine Angabe
**e.)** … zuversichtlich in Bezug auf Ihre Krankheit/ Gesundheit zu sein?	☐	☐	☐	☐	☐	☐
**f.)** … sich selbst zu helfen	☐	☐	☐	☐	☐	☐

Kontakt:
PD Dr. med. Stefanie Joos, Universitätsklinikum Heidelberg, Abteilung Allgemeinmedizin und Versorgungsforschung
Voßstr. 2, 69115 Heidelberg, Telefon: 06221/56-6263, Fax: 06221/56-1972

C. Fragen zu Ihrer Person

32.) Wie alt sind Sie? |_|_|_| **Jahre**

33.) Wie ist Ihr Geschlecht?

- ☐ männlich
- ☐ weiblich

34.) Wo ist Ihr Wohnort?

- ☐ Großstadt (ab 100.000 EW)
- ☐ Kleinstadt (bis 100.000 EW)
- ☐ Ländliches Umfeld (bis 15.000 EW)

35.) Welchen Familienstand haben Sie?

- ☐ ledig
- ☐ verheiratet / in Partnerschaft
- ☐ geschieden / getrennt lebend
- ☐ verwitwet

36.) Wie leben Sie?

- ☐ alleine lebend
- ☐ mit Partner/in lebend
- ☐ sonstiges

37.) Haben Sie ein Kind / Kinder?

- ☐ ja
- ☐ nein

38.) Welches ist Ihr <u>höchster</u> Schulabschluss *(bitte nur eine Angabe)***?**

- ☐ kein Schulabschluss
- ☐ Hauptschulabschluss / Volksschulabschluss
- ☐ Realschulabschluss (Mittlere Reife)
- ☐ Abitur /(Fach) Hochschulreife
- ☐ sonstiger Abschluss, und zwar:

39.) Sind Sie derzeit erwerbstätig?

- ☐ nein
- ☐ ja

<u>Wenn ja</u>,
- ☐.. mit mehr als 40 Stunden pro Woche
- ☐.. mit 20 - 40 Stunden pro Woche
- ☐.. mit weniger als 20 Stunden pro Woche

40.) Falls Sie <u>nicht</u> erwerbstätig sind, zu welcher Gruppe gehören Sie?

- ☐ Schüler/in
- ☐ Student/in
- ☐ Hausfrau/Hausmann
- ☐ Rentner/in, Pensionär/in
- ☐ arbeitslos
- ☐ sonstiges und zwar: |_|_|_|_|_|_|_|_|_|_|_|_|_|_|_|_|_|_|_|_|

41.) Wo sind Sie geboren?

- ☐ in Deutschland
- ☐ in einem anderen Land

42.) Welche Sprache sprechen Sie <u>überwiegend</u> zu Hause?

- ☐ deutsch
- ☐ eine andere Sprache
- ☐ keine Angabe möglich

Kontakt:
PD Dr. med. Stefanie Joos, Universitätsklinikum Heidelberg, Abteilung Allgemeinmedizin und Versorgungsforschung
Voßstr. 2, 69115 Heidelberg, Telefon: 06221/56-6263, Fax: 06221/56-1972

43.) Wie stark achten Sie auf Ihre Gesundheit?

überhaupt nicht ☐ ☐ ☐ ☐ ☐ ☐ ☐ sehr stark

44.) Rauchen Sie?

☐ nein ☐ ja ☐ gelegentlich ☐ Ex-Raucher

45.) Treiben Sie Sport? Wenn ja, wie häufig?

☐ nie ☐ 1 x pro Woche oder weniger ☐ mehrmals pro Woche ☐ täglich

46.) Haben Sie manchmal das Gefühl, dass Sie in einer ungewohnten Situation sind und nicht wissen, was Sie tun sollen?

sehr selten oder nie ☐ ☐ ☐ ☐ ☐ sehr oft

47.) Wenn Sie über Ihr Leben nachdenken, ist es dann sehr oft so, dass…

...Sie spüren, wie schön es ist, zu leben ☐ ☐ ☐ ☐ ☐ …Sie sich fragen, wieso Sie überhaupt leben

48.) Wie oft sind Ihre Gefühle und Gedanken ganz durcheinander?

sehr selten oder nie ☐ ☐ ☐ ☐ ☐ sehr oft

49.) Wie ist Ihr Versichertenstatus? *(Mehrfachantworten möglich)*

☐ gesetzlich krankenversichert ☐ private Zusatzversicherung
☐ privat krankenversichert ☐ Beihilfe (Beamtenrecht)

50.) Wie viele Kosten, die Sie aus eigener Tasche bezahlen, entstehen Ihnen durch Ausgaben für Ihre Gesundheit (z. B. Kosten für frei verkäufliche Medikamente, Individuelle Gesundheitsleistungen beim Arzt, Gesundheitskurse, Heilpraktikerbehandlungen etc.)?

ca. Euro |__|__|__| pro Monat

51.) Wie viel wären Sie bereit, im Monat zusätzlich für Ihre Gesundheit auszugeben?

ca. Euro |__|__|__| pro Monat

Haben Sie Kommentare zum Fragebogen oder möchten etwas ergänzen?
Dann tragen Sie es bitte hier oder auf einem zusätzlichen Blatt ein.

Vielen Dank für Ihre Mitarbeit !

Kontakt:
PD Dr. med. Stefanie Joos, Universitätsklinikum Heidelberg, Abteilung Allgemeinmedizin und Versorgungsforschung
Voßstr. 2, 69115 Heidelberg, Telefon: 06221/56-6263, Fax: 06221/56-1972
